# Design and Implementation of an Online Efficiency-Optimized Multi-Functional Compensator for Wind Turbine Generators

**DOI:** 10.3390/mi14101958

**Published:** 2023-10-20

**Authors:** Chao-Tsung Ma, Feng-Wei Zhou

**Affiliations:** Applied Power Electronics Systems Research Group, Department of EE, CEECS, National United University, Miaoli City 36063, Taiwan

**Keywords:** power semiconductor device (PSD), renewable energy (RE), wind turbine generator (WTG), energy storage unit (ESU), power quality (PQ)

## Abstract

In recent years, the penetration of wind power generation has been growing steadily to adapt to the modern trend of boosting renewable energy (RE)-based power generation. However, the dynamic power flow of wind turbine generators (WTGs) is unpredictable and can have a negative impact on existing power grids. To solve this problem efficiently, this paper presents a multifunctional WTG intelligent compensator (WTGIC) for the advanced power management and compensation of power systems embedded with WTGs. The proposed WTGIC consists of a power semiconductor device (PSD)-based bidirectional three-phase inverter module and an energy storage unit (ESU). In order to reduce system costs and improve reliability, efficiency, and flexibility, various control functions and algorithms are integrated via a modularized all-digital control scheme. In this paper, the configuration of the proposed WTGIC is first introduced, and then the operating modes and related compensation and control functions are addressed. An online efficiency optimization algorithm is proposed, and the required controllers are designed and implemented. The designed functions of the proposed WTGIC include high-efficiency charging/discharging of the ESU, real-time power quality (PQ) compensation, and high-efficiency power smoothing of the WTGs. The feasibility and effectiveness of the proposed WTGIC are verified using case studies with simulations in the Powersim (PSIM) environment and the implementation of a small-scale hardware experimental system with TI’s digital signal processor (DSP) TI28335 as the main controller.

## 1. Introduction

The trend of decarbonization and the gradual decrease in conventional fossil energy sources make the development of RE-based generation an urgent task [1]. Among various RE sources, wind energy is inexhaustible and inexpensive, which makes it one of the most promising RE sources [2,3,4,5]. Wind power generation technology is relatively mature and can generate a large amount of electricity. However, the real-time output quantity of wind power is naturally unpredictable and may lead to severe PQ problems. As a result, the smart microgrid (MG) concept can be used to construct more flexible power management in power systems embedded with WTGs. The operation of a smart MG is different from that of a conventional standalone power system and can control its connection and disconnection to larger external power systems when needed. The advantages of smart MGs include lower costs in new installments, easy integration of RE-based distributed generation (DG), and improved PQ, reliability, and efficiency. Therefore, in line with the low-carbon development trend, the integration of DG, MGs, energy storage systems (ESSs), and adequate power system compensators with advanced power management techniques is vitally needed.

Common PQ problems in MGs include voltage sag, voltage swell, and harmonic distortion. During voltage sag, reactive power support capability is necessary to maintain the system voltage and thus avoid possible damage to the power system and losses of electricity users. Via capacity configuration and the adoption of a concentrated reactive power compensator, He et al. [6] improved the LVRT capability of a wind farm. Static VAR generators (SVGs) and WTs were adopted as the compensator and main reactive current sources, respectively. A distribution static compensator (D-STATCOM) and bridge-type fault current limiter (BFCL) were used in [7] to reduce the negative effect of harmonic filter parameter variations, which are due to aging or thermal drift, on LVRT capability. The quantum field theory (QTF) approach was adopted as the control scheme. In [8], it was pointed out that it was difficult to access offshore doubly fed induction generators (DFIGs), so it was very important to avoid damages and shutdowns of an offshore WTG. A series voltage compensator (SVC) with minimized components was used to control the generator stator flux. The design reduced the stator and rotor currents and rotor voltage. A model reference adaptive control (MRAC) was proposed in [9] for a STATCOM to improve the LVRT capability of a grid-connected WTG. Compared with genetic-algorithm-based proportional–integral (PI) controllers, the proposed control exhibited better efficiency. A coordinated current control scheme was proposed in [10] to deal with grid faults. The proposed control strategy maintained the synchronization stability of both the WTG and the STATCOM. In addition, reactive currents could be produced with the synchronized STATCOM to meet the grid code.

Zhu et al. [11] focused on damping sub-synchronous oscillation (SSO) by using a static synchronous series compensator (SSSC)-based hybrid compensator. The results showed that the transmission capacity of the system was increased, and the supplementary control effectively improved the stability of the system without a negative effect on the compensator performance. A wide-area damping controller was designed in [12] to increase oscillation damping using a STATCOM and network predictive control (NPC). The simulation results showed an improved LVRT capability, reduced low-frequency oscillations, satisfactory compensation for constant and random time delays, robust control, and superior performance to those of conventional methods. In [13], a STATCOM was used to connect two power systems to an integrated onshore–offshore wind farm. A lead-lag power oscillation damping controller for the STATCOM was designed. The results showed the improved stability of the entire system. A novel interval type II fuzzy control system (IT2-FCS) for PI controller tuning was proposed in [14] for bus voltage stabilization. A fast and stable system response was realized with IT2 fuzzy rules. The results showed a better performance than those of conventional PI and type I FCS methods. To improve the PQ of a single-phase grid connected to a WTG and a solar photovoltaic (PV) array, a control technique based on a third-order signal integrator was used in [15] to extract double fundamental signals of load current and fundamental signals of grid voltage. Using the extracted signals, the interface converter operated as a D-STATCOM under various wind and solar irradiance conditions to maintain good PQ. The effectiveness of using a STATCOM for sub-synchronous resonance (SSR) damping in a DFIG-based wind farm was studied in [16]. Via various simulation cases in the PSCAD/EMTDC environment, the proposed method was proven to be practical as well as effective. A control scheme employing both a thyristor-controlled series compensator (TCSC) and STATCOM dual-loop feedback control was proposed in [17] to handle voltage stability problems in grid-connected WTGs. Simulations in the MATLAB/Simulink environment proved that the proposed method outperformed only the TCSC and only the STATCOM in voltage recovery.

Regarding the aspect of the optimal design of MGs embedded with WTGs, Bai et al. [18] proposed an AC optimal power flow (RACOPF) model based on the affinely adjustable robust OPF (AAROPF) model, which was developed for power networks with RE sources. Three test systems were numerically analyzed and yielded better performances than those of conventional DC OPF models. In [19], WT operating traits and frequency regulation characteristics were explored, and a simplified dynamic power flow (DPF) algorithm was proposed for wind-power-integrated power systems. The performance of the proposed method under different levels of wind power penetration was verified with the IEEE 30-bus system. A fuzzification-based multi-objective stochastic OPF (SOPF) problem was explored in [20], where operation cost, voltage stability, and emission effects were considered. The technique of fuzzification normalized all objective functions and found the best solution. A line voltage stability index (LVSI) for voltage collapse detection was also proposed. A modified WTG test system, IEEE 39-bus, was used to validate the proposed method. Rahmani et al. [21] presented a multi-objective information-gap decision theory (IGDT)-based AC OPF model for simultaneous optimization under the conditions of varying load demands and wind-power-related uncertainties. An effective directed search domain (DSD)-based multi-objective solution method was also proposed. The IEEE 118-bus test system was used to test the proposed model and approach, which were proven to be effective. A quasi-Monte Carlo simulation (QMCS)-based probabilistic OPF (POPF) technique was applied to probabilistic power flow (PPF) in [22]. The wind speed-dependent model and forecast errors were based on a copula function. QMCS reduced the computation complexity. A modified IEEE 118-bus power system connected to wind farms was used to verify the accuracy and efficiency of the proposed method.

Theoretically, there are a lot of advantages of employing MGs and DGs, but there are also many challenges related to operation, protection, system optimization, and control when various DGs are connected to an MG, and it is, in fact, not easy to deal with all these problems simultaneously. In view of this, this paper proposes a power-converter-based WTG intelligent compensator (WTGIC) and related integrated control schemes and verifies its feasibility and effectiveness. The proposed WTGIC is an implementation of the optimal system design concept that takes into account the cost, efficiency, functional flexibility, and operating characteristics of a WTG. With a properly designed control algorithm, the proposed WTGIC is a power semiconductor device (PSD)-based, high-performance WT compensation device that integrates a number of compensation capabilities, output power smoothing, point of common coupling (PCC) PQ management, and high-efficiency charge/discharge control of the energy storage unit (ESU). For existing WTGs that have already been installed, and whose internal control structure cannot be modified, the proposed WTGIC adopts an external high-efficiency, multi-functional integrated modular system configuration to perform various functions. The proposed system has three major features: (1) a modular design to facilitate a flexible and expandable system capacity; (2) the programmable digital control scheme enables multiple integrated control functions, and (3) the parallel connection of multiple converters enables the online efficiency optimization feature.

Following the introduction section, Section 2 introduces the proposed WTGIC configuration and designed operating modes. Section 3 explains the design of the WTGIC converters and the development of the required controllers and algorithms, where mathematical models of the converters and controllers are derived. Section 4 carries out PSIM simulation studies, where the three operating modes in the WTGIC are all presented. Section 5 carries out the small-capacity experimental hardware implementation of the proposed WTGIC system. Finally, the major work of this paper is summarized in Section 6.

## 2. WTGIC System Configuration and Operating Modes

**Figure 1:**
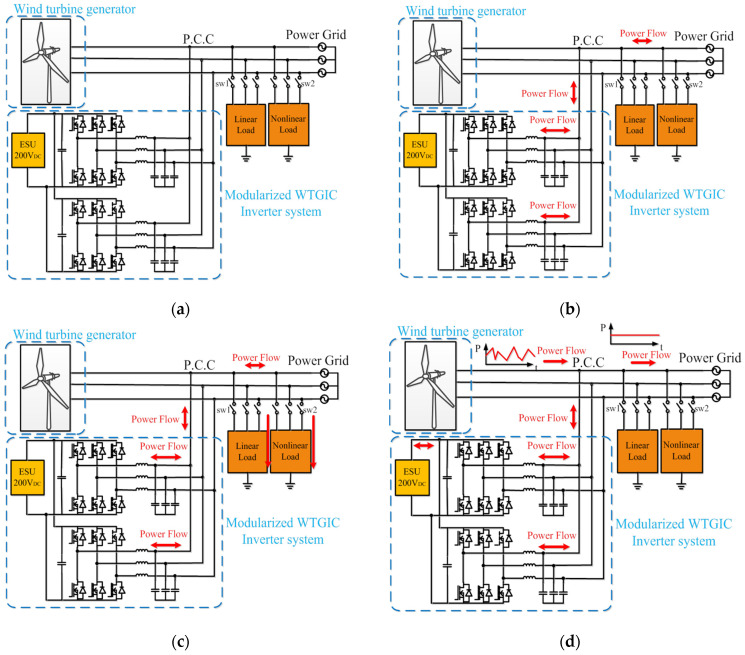
Proposed modular WTGIC: (**a**) system configuration, (**b**) operating mode 1, (**c**) operating mode 2, and (**d**) operating mode 3.

As shown in Figure 1a, the system configuration of the proposed WTGIC is a modular design with two identical three-phase inverter hardware units and an ESU. When the WTGIC performs the designed advanced control functions, a real-time optimal operating efficiency algorithm is used to simultaneously control two inverters. The designed WTGIC system can regulate its bidirectional current injecting into the system to which the WTGIC is connected to meet a variety of system compensating functions and specifications and adjust the capacity dispatching of the inverters and operating modes in real time depending on the need of the power grid operation. In this study, three typical operating modes and their power flow paths are shown in Figure 1b–d, respectively. The proposed system employs a 5 kW WTG module, and two 1 kVA three-phase inverters are connected in parallel to form the modular WTGIC inverter system. In the designed four operating modes, a real-time efficiency optimization strategy (REOS) is applied so that the modular inverter system is able to improve the system efficiency.

### 2.1. Operating Mode 1: Independent Charging/Discharging of ESU

In this mode, it is assumed that the state of charge (SOC) of the ESU is too low or too high to support the WTGIC’s functional operations and thus requires the regulation of the charging or discharging of the ESU. In addition, the charging or discharging of the ESU can be performed in some typical cases, e.g., automatic frequency control, damping control, and the emergency power dispatching of the power grid when an emergency occurs.

### 2.2. Operating Mode 2: PQ Improvement

In general, power systems cannot avoid connecting with various nonlinear loads, which are likely to cause system interference or PQ degradation. In severe cases, a power system may enter an unstable state, causing unpredictable damage. In this mode, sw_1_ and sw_2_ are on (see Figure 1c). When the load is an inductive or capacitive load, the power factor (PF) can be adjusted via reactive power compensation, thereby improving the power transmission efficiency. When there are harmonic loads, the harmonic current can be also compensated with the proposed WTGIC.

### 2.3. Operating Mode 3: WTG Power Smoothing

During normal operation, the output power of a WTG operated in maximum power tracking control mode may change drastically within a short period of time, significantly reducing the voltage stability and reliability of a grid if not regulated. In this operating mode, the WTGIC can achieve power smoothing through power dispatch and improve the fluctuation in the output power of WTG via the real-time charging/discharging of the ESU. It should be noted that this mode requires a moderate SOC of the ESU. If the SOC is too high or too low, over-charging or over-discharging may occur.

## 3. Design of WTGIC Power Converters and Development of Required Controllers and Algorithms

### 3.1. WTGIC Power Converters

**Figure 2:**
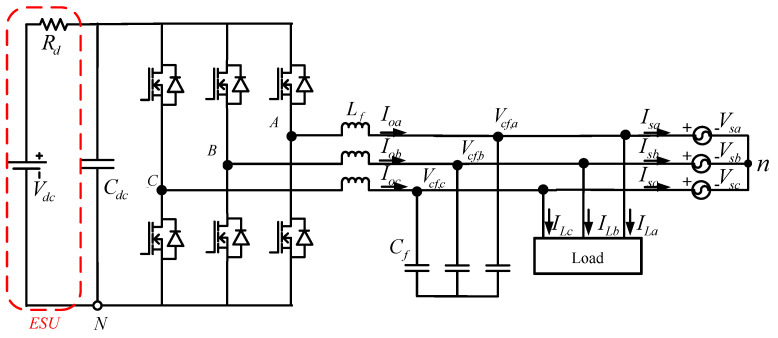
WTGIC single-module three-phase inverter connected to a grid and a load.

The hardware circuit configuration of a single-module WTGIC is shown in Figure 2, where *V_dc_* denotes the DC link voltage; *R_d_* denotes the internal resistance of the ESU; *C_dc_* denotes the DC link capacitor; *N* denotes the inverter ground; *A*, *B*, and *C* denote the inverter switching points; *L_f_* denotes the filter inductor; *I_oa_*, *I_ob_*, and *I_oc_* denote the inductor currents; *V_cf,a_*, *V_cf,b_*, and *V_cf,c_* denote the filter capacitor voltages; *C_f_* denotes the filter capacitor; *I_La_*, *I_Lb_*, and *I_Lc_* denote the load currents; *I_sa_*, *I_sb_*, and *I_sc_* denote the grid currents; *V_sa_*, *V_sb_*, and *V_sc_* denote the grid voltages; and *n* denotes the grid ground. The inverter’s three-phase output is connected to the grid via a low-pass filter (LPF). When using the three-phase inverter as a WTGIC, it is necessary to regulate the system’s bidirectional P and Q. In order to ensure stable operation, a dual-loop control architecture is adopted, where the inner loops perform the current control, and the outer loops perform the tracking control of the bidirectional P and Q commands. An ESU is placed on the DC link of a WTGIC for bidirectional active power feeding.

### 3.2. Real-Time Efficiency Optimization Strategy and Control

**Figure 3:**
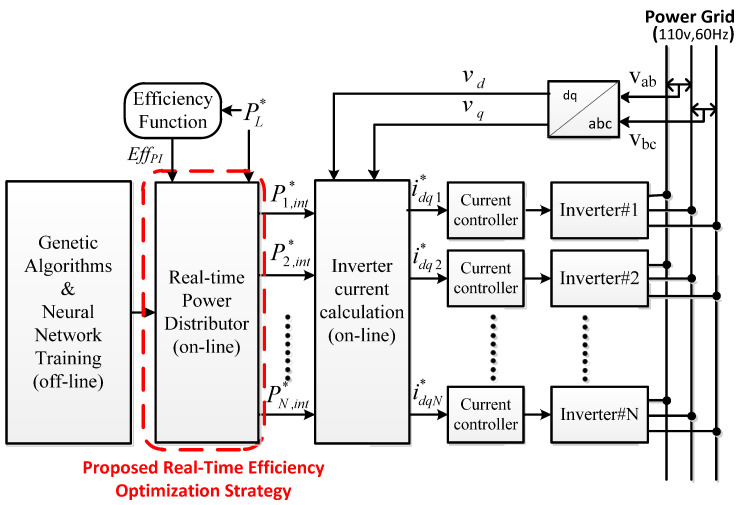
Design process and signal path of the proposed REOS.

At present, most of the control strategies for parallel inverters in the literature are for suppressing the influence of the circulating current via the average current sharing strategy (ACSS). However, the disadvantage of the ACSS is its extremely low conversion efficiency, especially under light-load conditions. In order to improve the conversion efficiency under light-load conditions, this paper proposes a real-time efficiency optimization strategy (REOS) for the proposed WTGIC using a modular three-phase inverter system. The system architecture of the REOS is shown in Figure 3, where *V_ab_* and *V_bc_* denote the grid line voltages, *v_d_* and *v_q_* denote the grid dq-axis voltages in a synchronous reference frame (SRF), *i_dq_*_1_ to *i_dqN_* with asterisks denote the dq-axis current commands, *P*_1,*int*_ to *P_N,int_* with asterisks denote the optimal dispatching power commands, *P_L_* with an asterisk denotes the WTGIC’s real power command, and *Eff_PI_* denotes the best efficiency of the WTGIC’s parallel inverters. The REOS algorithm must determine the number of inverter modules activated for each operation and the power distribution factor of each module within a given power range. Assuming that each inverter has the same power specification and efficiency feature, based on the conversion efficiency curve of a single three-phase inverter, the optimal conversion efficiencies and power distribution factors of the parallel inverters for several working points are calculated offline using a genetic algorithm. Then, a neural network (NN) is trained to learn the best distribution factors according to the power command for the WTGIC and the corresponding best efficiency of the WTGIC’s parallel inverters (*Eff_PI_*) at the selected working points. Finally, the trained NN can be used online, as shown in Figure 3, to act as a real-time optimal power distributor to achieve the best overall conversion efficiencies at any operating points within the maximum rated power of the WTGIC.

**Figure 4:**
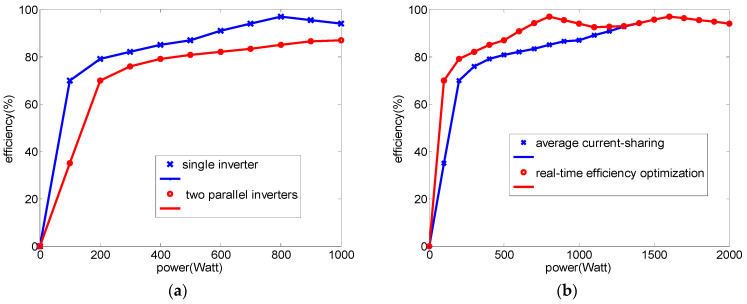
Efficiency comparison: (**a**) 1 kW single inverter vs. two 1 kW inverters in parallel with ACSS; (**b**) proposed two 1 kW inverters module adopting different control strategies (REOS and ACSS).

To support the design concept of the REOS, some efficiency studies with experimental tests were carried out. In the experiment, two 1 kVA inverters were connected in parallel. The actual measurements show that the efficiency of a single inverter under a light load was extremely low; however, multiple inverters connected in parallel and adopting the ACSS resulted in an even lower efficiency under a light load. The power conversion efficiency curves of a single inverter and a developed two 1 kVA parallel-connected inverters module between 100 W and 1 kW are shown in Figure 4a. It can be clearly seen that the conversion efficiency of the parallel module adopting the ACSS is lower than that of the single inverter at all operating points. Figure 4b shows the efficiency curves of the proposed inverter module adopting the ACSS and REOS at different operating points. It can be seen that when the power level is greater than 1.3 kW, the efficiencies of the two strategies are almost the same. However, when the power level is less than 1.3 kW, the proposed REOS can effectively improve the conversion efficiency compared with the ACSS. Therefore, the REOS is feasible for improving the efficiency of multi-inverter systems.

#### 3.2.1. WTGIC Inner Loops—Inductor Current Control

**Figure 5:**
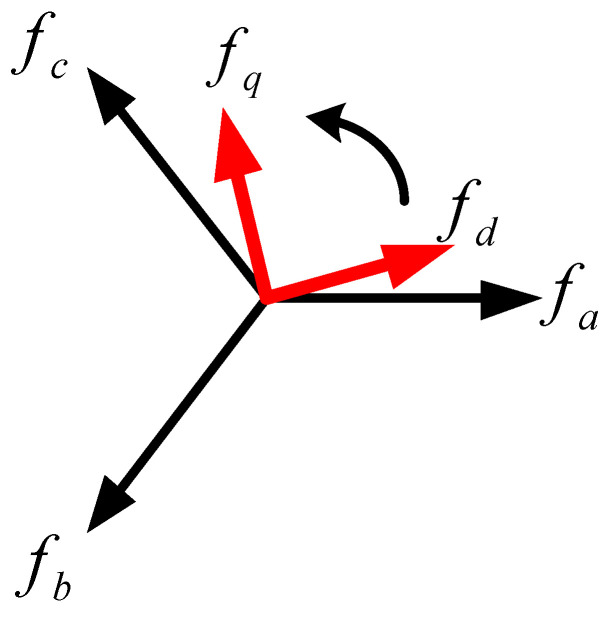
The transformation of abc-axis signals into dq-axis signals in SRF.

In a three-phase inverter, voltages and currents are coupled to each other. If a three-phase Cartesian coordinate is used to build the system, it is more complicated when it comes to the calculations and the design of the controllers. Therefore, in order to simplify the analysis and controller design, signals in the abc-axes are usually converted into αβ-axis or dq-axis equivalents via frame conversion. The αβ-axis signals in a static frame are all AC terms, where general controllers yield poor performances. Therefore, a more robust controller is needed for an acceptable control performance if an αβ-coordinate is adopted. On the other hand, the dq-axis signals in a synchronous reference frame (SRF) are all DC terms, which can be well controlled using a general type-II controller. Figure 5 conceptually shows the transformation of the abc-axis signals into dq-axis signals in an SRF. When using this method, it is required to employ a phase-lock loop (PLL) to provide a synchronization phase angle and adopt a control design with a decoupling function, which is computationally intensive. Nevertheless, recent developments have made it possible for DPSs to perform complex mathematical calculations and solve problems that require large calculations. The dual-loop controller used in this paper was built using feedforward control to adjust the direction and magnitude of the inverter current output. To obtain the mathematical model of the inductor currents of the three-phase inverter, Kirchhoff’s current law and the SRF theory were used to obtain the dq-axis inductor current equations:

(1) $$I_{o,d}=\frac{1}{SL_{f}}(-V_{d}-\omega L_{f}I_{o,q}+K_{pwm}V_{con,d});$$

(2) $$I_{o,d}=\frac{1}{SL_{f}}(-V_{d}-\omega L_{f}I_{o,q}+K_{pwm}V_{con,d}),$$

where *K_pwm_* equals *V_dc_*/2*V_tri_*, and *V_con,d_* and *V_con,q_* denote the dq-axis control signals. Consequently, the control equations with control signals as outputs are as follows:

(3) $$V_{con,d}=K_{pwm}{}^{-1}[L_{f}\frac{dI_{o,d}}{dt}+\omega L_{f}I_{o,q}+V_{d}];$$

(4) $$V_{con,q}=K_{pwm}{}^{-1}[L_{f}\frac{dI_{o,q}}{dt}-\omega L_{f}I_{o,d}+V_{q}].$$

**Figure 6:**
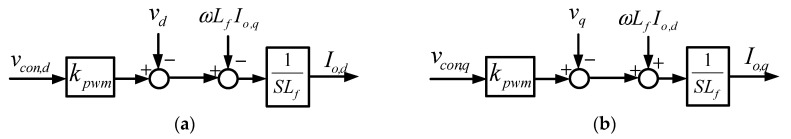
Mathematical models of the grid converter inductor currents: (**a**) d-axis; (**b**) q-axis.

**Figure 7:**
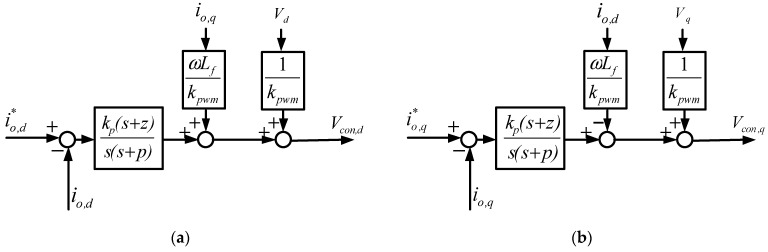
Mathematical models of grid converter inductor current controllers: (**a**) d-axis; (**b**) q-axis.

Finally, the qd-axis models of the WTGIC converter can be established according to (1) and (2), as shown in Figure 6, and the qd-axis models of the inverter’s inner loop inductor current controllers can be established according to (3) and (4), as shown in Figure 7. As shown in Figure 7, two type-II compensators were adopted for the qd-axis inductor current controllers. The crossover frequency of the inner loops was designed at 1/10 of the switching frequency of 20 kHz, which is 12,566.37 rad/s, the zero was designed at 4188.8 rad/s, and the pole was designed at 43,982.3 rad/s to achieve a phase margin of 56 degrees. As a result, the controller transfer function was designed as follows:

(5) $$G_{i}(s)=\frac{k_{1}(s+z)}{s(s+p)}=\frac{12.3986(s+4188.8)}{s(s+43,982.3)}$$

Using the proposed REOS for the WTGIC’s modular control can achieve stable execution using the least amount of control signals. The developed intelligent optimal distributor can optimally distribute currents according to the WTGIC’s total power/current command so that most of the modules can operate at full load where the efficiency is high.

#### 3.2.2. WTGIC Outer Loops—P and Q Control

**Figure 8:**
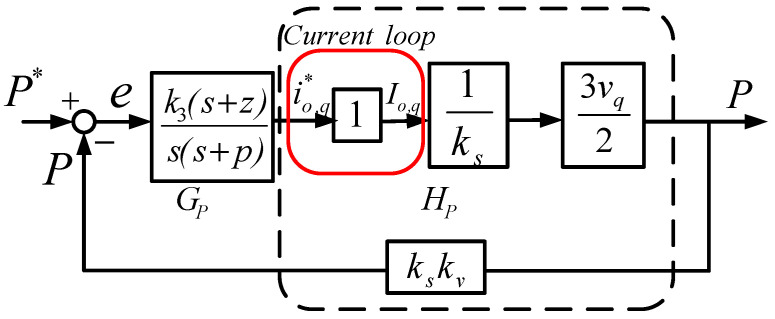
Mathematical model of active power control loop.

**Figure 9:**
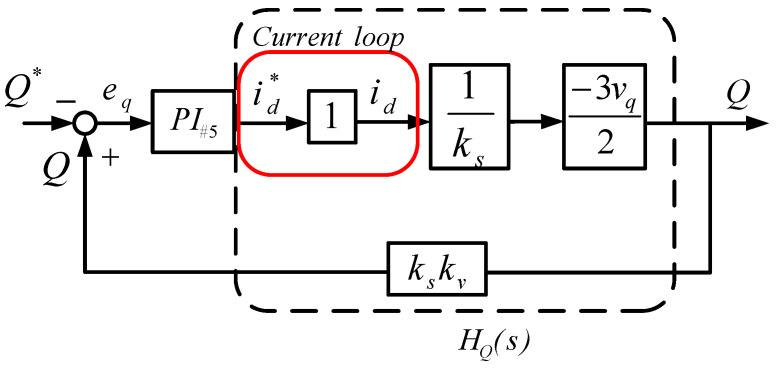
Mathematical model of reactive power control loop.

The dq-axis models of the WTGIC’s active/reactive power controller using a type-II compensator are shown in Figure 8 and Figure 9. It is assumed that the bandwidths of the current control loops are much larger than those of the active/reactive power control loops.

The crossover frequency of the outer loops was designed at 1/3 of that of the inner loops, which was 4188.79 rad/s, the zero was designed at 2094.39 rad/s, and the pole was designed at 8377.57 rad/s. As a result, the controller transfer function was designed as follows:

(6) $$G_{P,Q}(s)=\frac{k_{3}(s+z)}{s(s+p)}=\frac{1.1974(s+2094.39)}{s(s+8377.58)}.$$

Figure 9 shows an open-loop Bode plot of the active/reactive power control loops. The phase margin is 127 degrees.

#### 3.2.3. WTGIC’s Harmonic Current Compensation Technique

**Figure 10:**
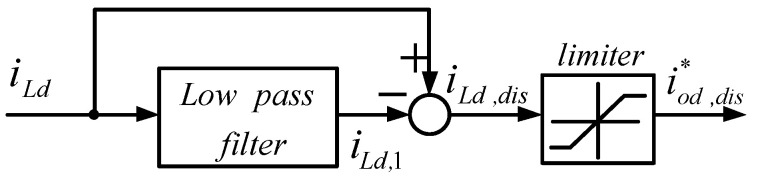
Calculation of d-axis harmonic compensation command.

**Figure 11:**
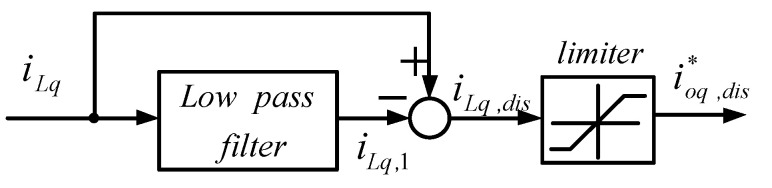
Calculation of q-axis harmonic compensation command.

In this paper, an LPF was used to obtain the fundamental components of the load currents, and the fundamental waves were then subtracted from the complete load currents to achieve the harmonic components required for compensation, as shown in Figure 10 and Figure 11.

#### 3.2.4. Complete Control Architecture

**Figure 12:**
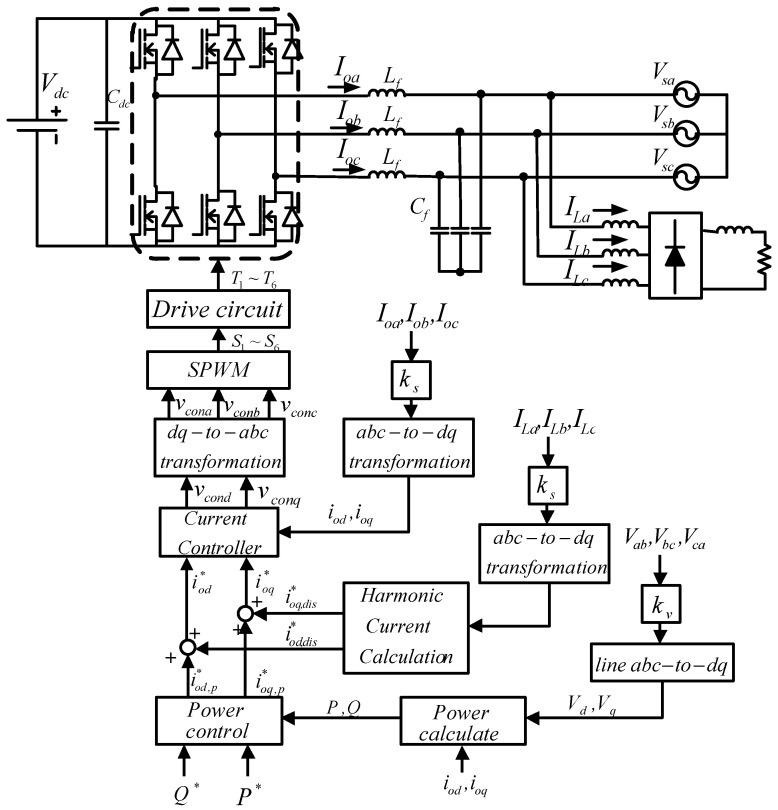
Complete WTGIC control system (single module).

Figure 12 shows the complete WTGIC system proposed in this paper, including a grid-tie three-phase inverter, an ESU installed at the DC side, and the dual-loop controller designed for real-time regulating the active and reactive powers and inductor currents.

## 4. System-Wide Simulation of Proposed WTGIC

**Figure 13:**
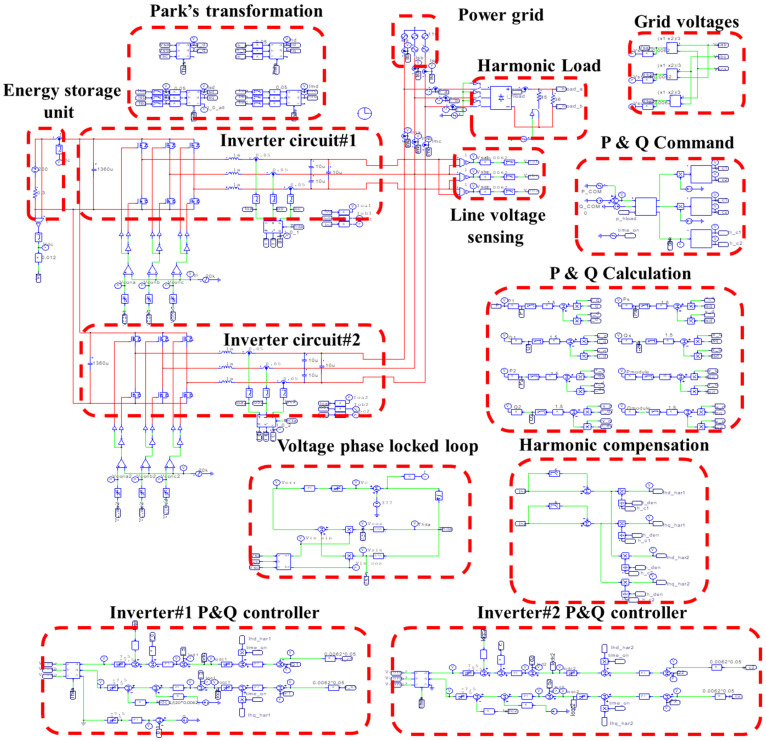
PSIM simulation model of the proposed WTGIC system.

In this paper, a reliable power electronics simulation software, PowerSIM (PSIM Ver. 9.0) was used for the case simulation. The simulated modular WTGIC system configuration is shown in Figure 13. In this section, the proposed WTGIC’s three typical operating modes are simulated and discussed.

### 4.1. Operating Mode 1: Independent Charge/Discharge of ESU

**Figure 14:**
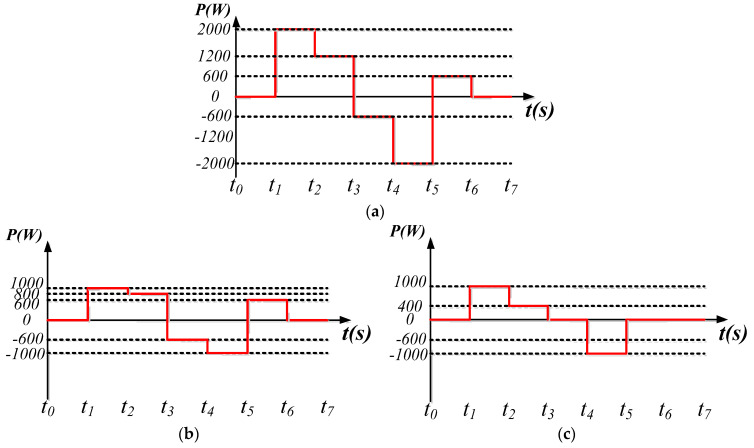
Operating mode 1: power variation sequence diagrams of: (**a**) WTGIC system; (**b**) inverter module #1; and (**c**) inverter module #2.

**Figure 15:**
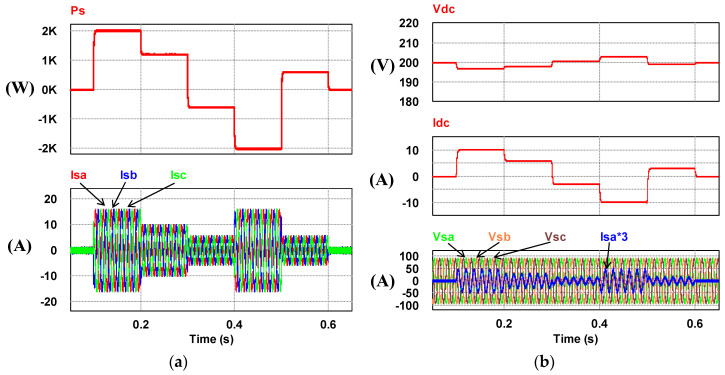

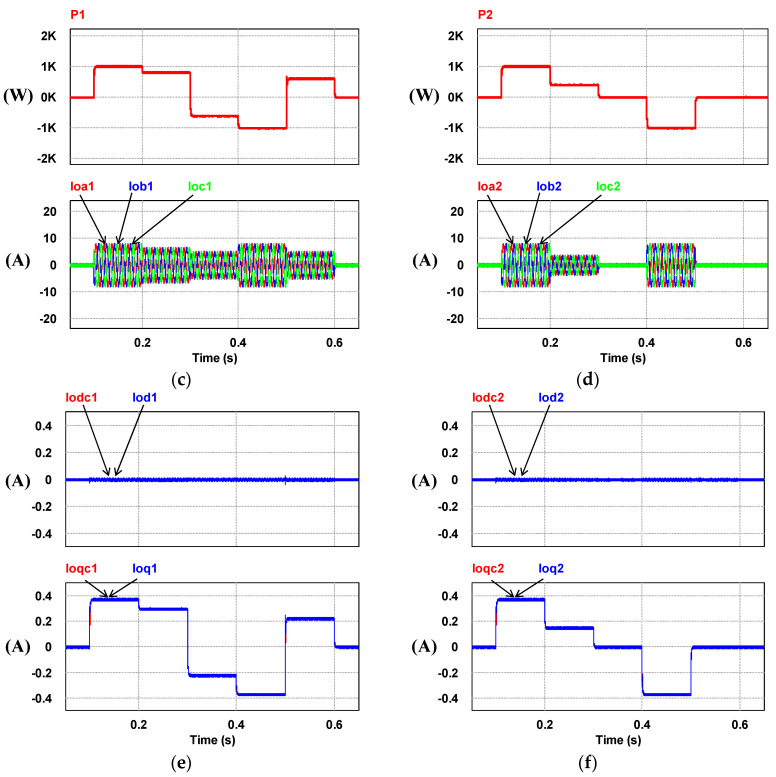
Operating mode 1: (**a**) The grid power and currents; (**b**) Parameters of the modular inverter; (**c**) Inverter module #1; (**d**) Inverter module #2; (**e**) The d-q current command and response of inverter module#1; (**f**) The d-q current command and response of inverter module#2.

In this mode, the WTGIC’s inverter unit and the power grid are activated. Figure 14 shows the power variation scenarios of the WTGIC’s modular inverter, inverter module #1, and inverter module #2, respectively. Figure 15 shows the simulation waveforms of the modular inverter. The system accurately distributes power commands and achieves stable results with the designed controllers.

### 4.2. Operating Mode 2: PQ Improvement

**Figure 16:**
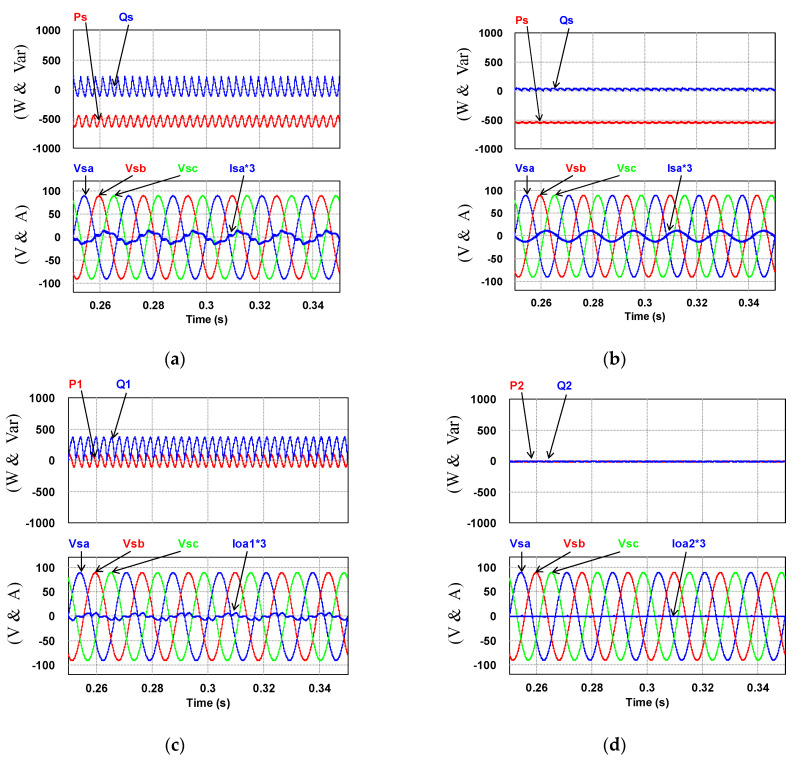
Operating mode 2: (**a**) the load powers/grid voltages and phase-a current (Ps = −676 W; Qs = −48 Var) before WTGIC being activated; (**b**) grid powers/grid voltages and phase-a current (Ps = −543 W; Qs = 0 Var) after WTGIC being activated; (**c**) inverter module #1 powers/AC voltages and phase-a current (P1 = 133 W; Q1 = 48 Var); and (**d**) inverter module #2 powers/AC voltages and phase-a current (P2 = 0 W; Q2 = 0 Var).

In this mode, the inverter unit, the load unit, and the power grid are activated. The load consists of a resistor and an inductance in series (RL) and a rectifier representing the harmonic load. Four cases were planned for this mode. The system parameters of various load conditions are arranged in Table 1; however, this paper only presents case 3 of the four cases shown in Table 1 in order to limit the number of pages. The simulation results showing the typical waveforms are shown in Figure 16.

### 4.3. Operating Mode 3: WTG Power Smoothing

**Figure 17:**
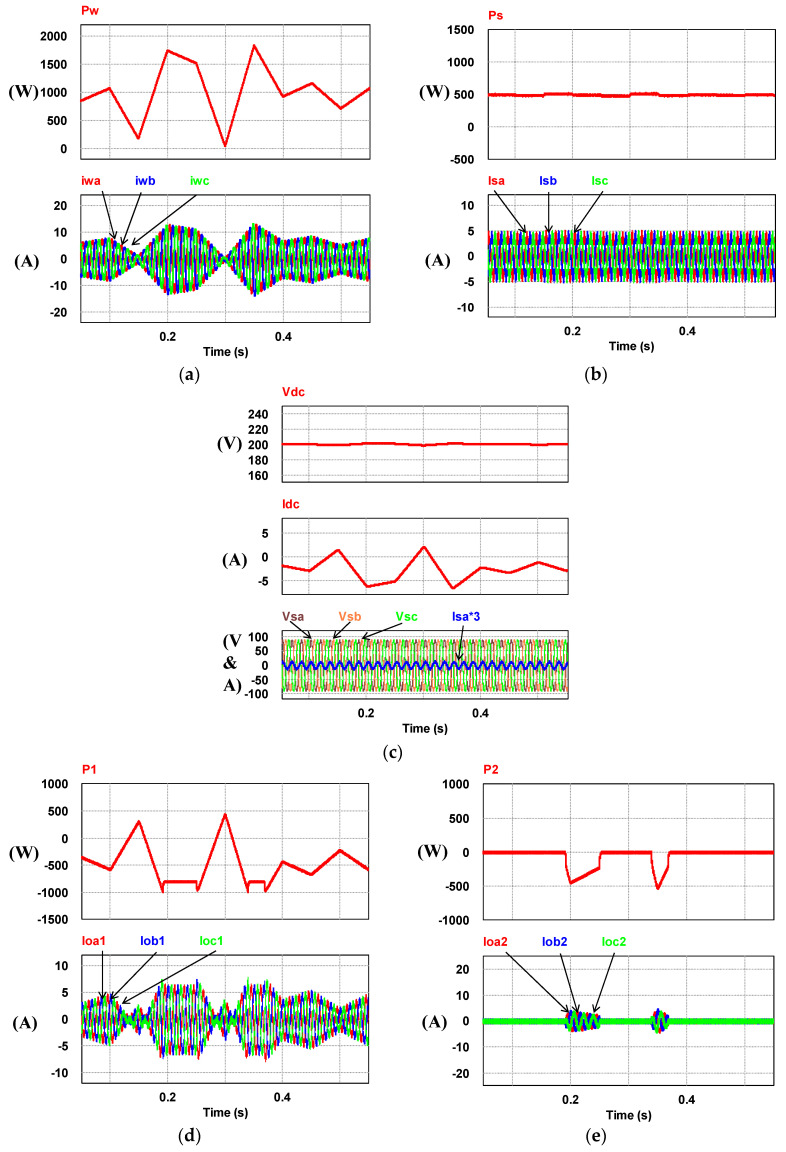
Operating mode 3: (**a**) WTG output power and AC currents; (**b**) grid power and three-phase AC currents; (**c**) WTGIC’s total DC and AC voltages and currents; (**d**) WTGIC’s inverter module #1 power and currents; and (**e**) WTGIC’s inverter module #2 power and currents.

In this operation mode, the WTG unit, the inverter unit, and the power grid are all activated. WTG output power varies between 0 W and 1840 W. The target power of the WTG fed into the grid is fixed at 500 W. Figure 17 shows the related simulation waveforms. It can be seen that regardless of the variation in the WTG output power, as shown in Figure 17a, the WTIGIC system can be controlled to achieve a dispatched power of 500 W feeding back to the grid, as shown in Figure 17b.

## 5. Small-Capacity Hardware Implementation of Proposed WTGIC

**Figure 18:**
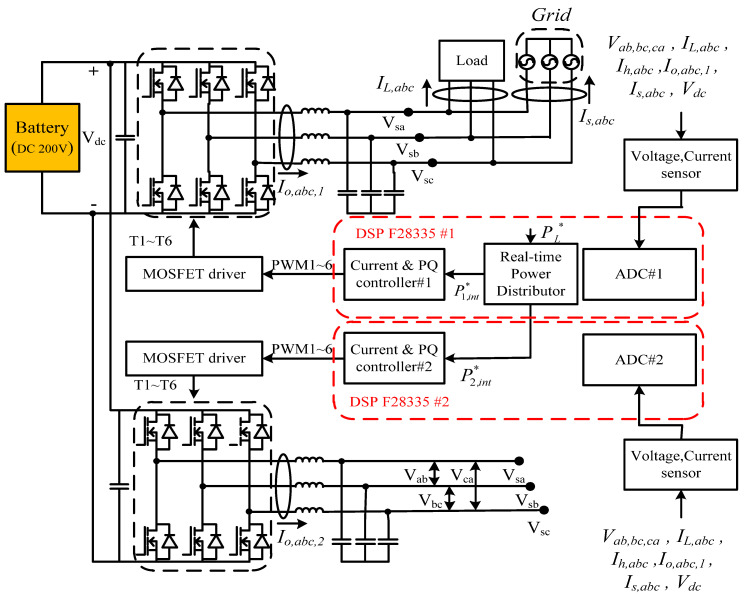
Schematic configuration of proposed modular WTGIC hardware and its signals.

**Figure 19:**
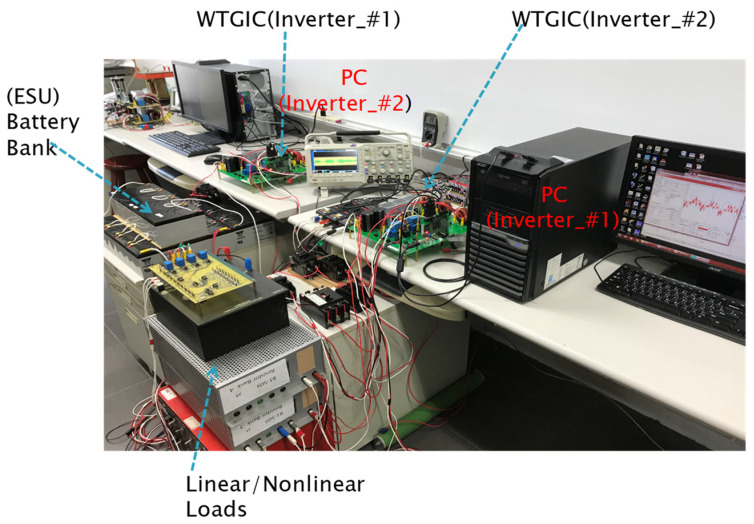
Photo of proposed small-capacity DSP-controlled WTGIC system hardware implementation.

**Figure 20:**
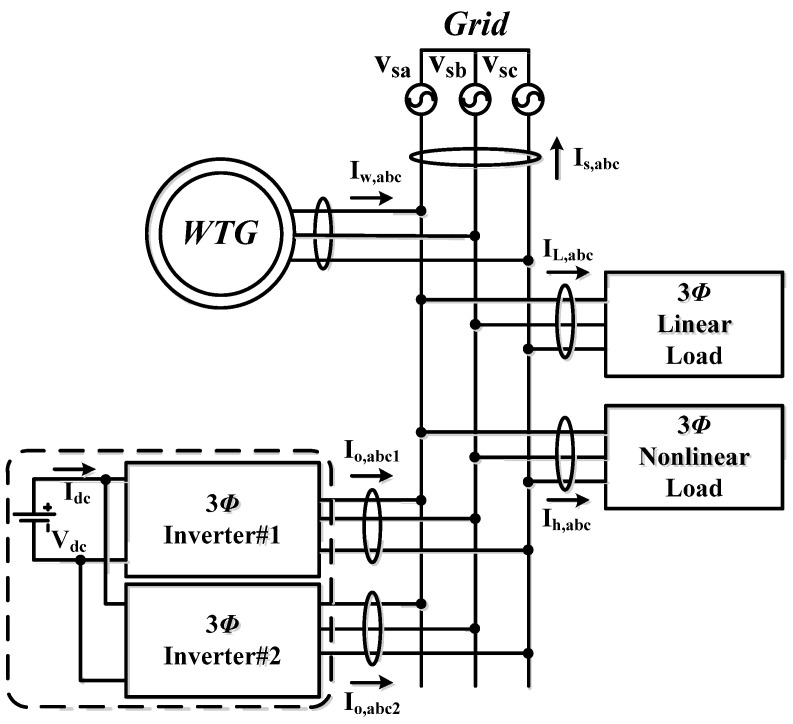
Measurement points of the hardware setup in all test cases.

The hardware experimental system in this paper adopted a WT emulator in the laboratory and an experimental 200 V, 10 A battery bank as the WTGIC’s ESU. Considering the voltage specification of the existing laboratory battery, the three-phase AC system representing the power grid was designed at 110 V; the DC and AC voltages of the modular three-phase inverter were designed at 200 V and 110 V/60 Hz, respectively; and each single module inverter was rated at 1 kVA, using six power semiconductor devices rated at 500 V/26 A. The bidirectional inverters were developed using two Texas Instrument DSP F28335 devices as the main controllers. Other experimental units included a personal computer (PC), self-made control interface circuits, voltage and current signal sensing and adjustment modules, driving and protection modules, various DC power modules, and a three-phase isolated transformer. Figure 18 shows the schematic configuration of the developed hardware and signals of the WTGIC. Figure 19 shows a photograph of the WTGIC hardware and the experimental environment. The operating conditions and system parameters of the cases are the same as those used in the simulation studies. Figure 20 shows the measurement points of the voltage and current waveforms.

### 5.1. Operating Mode 1: Independent Charge/Discharge of ESU

**Figure 21:**
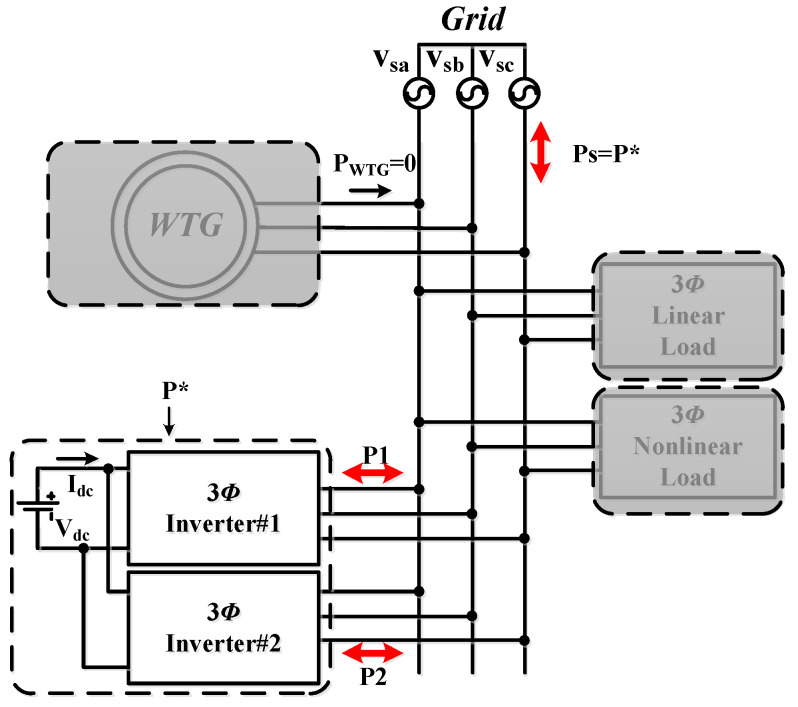
Operating mode 1: operation and power flow.

**Figure 22:**
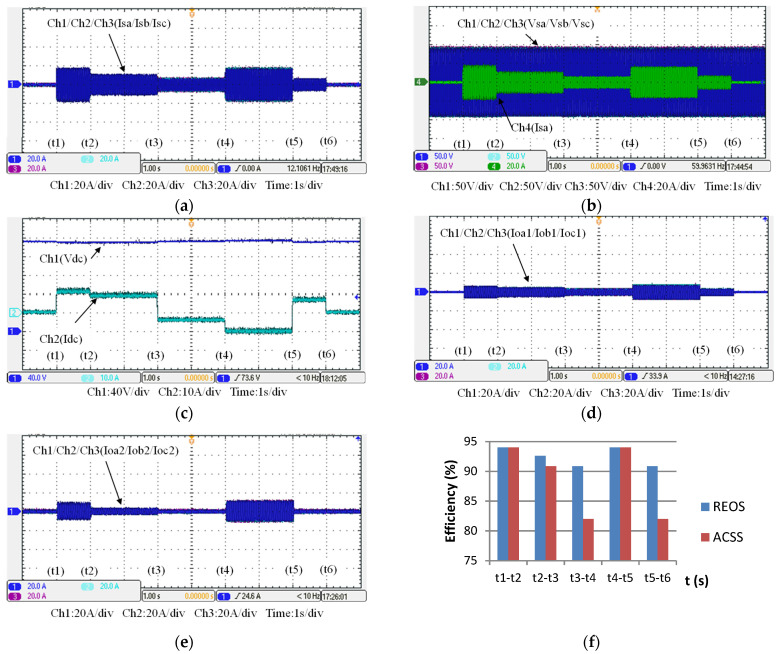
The measured results of operating mode 1: (**a**) WTGIC’s AC currents; (**b**) the grid voltages and phase-a current; (**c**) WTGIC’s DC voltage and current; (**d**) inverter module #1 AC currents; (**e**) inverter module #2 AC currents; and (**f**) WTGIC’s efficiency comparison between different control strategies.

This case is implemented to verify the simulation results of case 1. Figure 21 shows the operation and power flow of this operating mode. A set of typical measured results of this operation is presented in Figure 22.

### 5.2. Operating Mode 2: PQ Improvement

**Figure 23:**
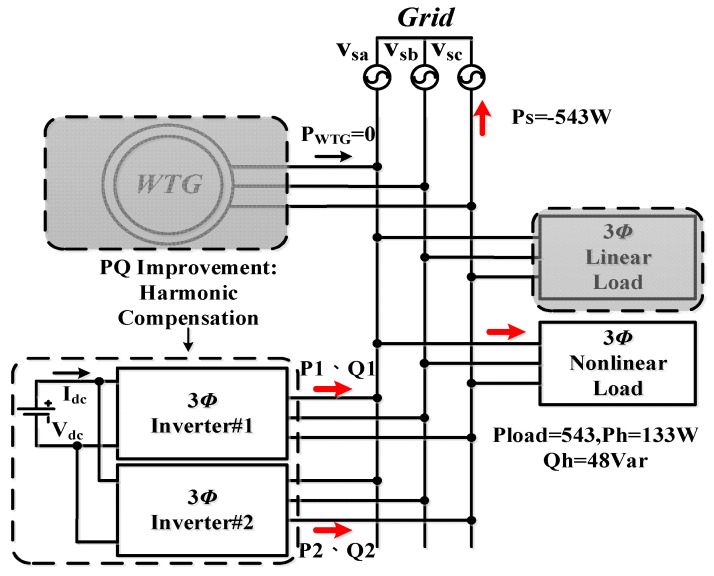
Operating mode 2: operation and power flow.

**Figure 24:**
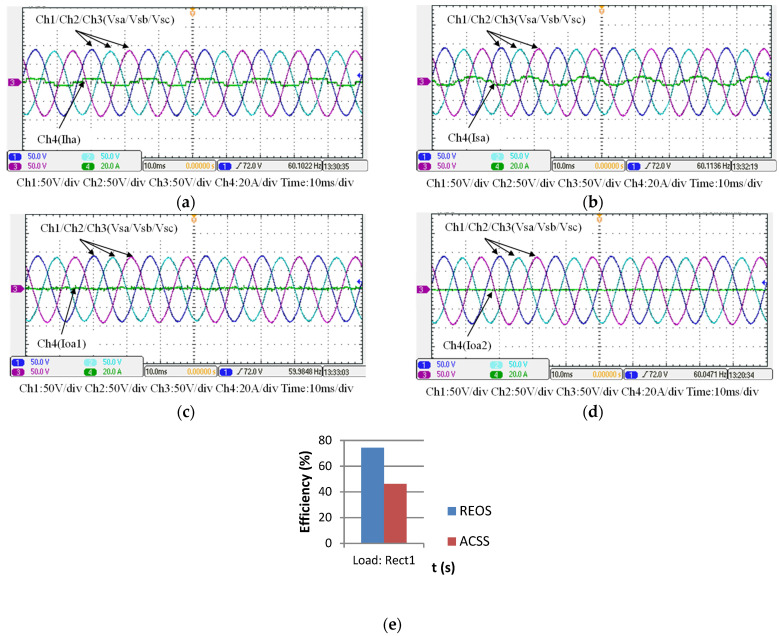
Operating mode 2: (**a**) the load voltages and phase-a current (Pload = 676 W; Qload = 48 Var); (**b**), the grid voltages and phase-a current (Ps = −543 W; Qs = 0 Var) after WTGIC being activated; (**c**) inverter module #1 AC voltages and phase-a current (P1 = Ph = 133 W; Q1 = 48 Var); (**d**) inverter module #2 voltages and phase-a current (P2 = 0 W; Q2 = 0 Var); and (**e**) WTGIC’s efficiency comparison between different control strategies.

This case is implemented to verify the simulation results of case 2. A steady-state AC/DC rectifier is used as the load in this mode. The control commands are set as follows: the WTGIC’s harmonic compensation function is activated with active power command P* = 0 W and reactive power command Q* = 48 Var. Figure 23 shows the system operation and power flow of this operating mode. Typical results are presented in Figure 24.

### 5.3. Operating Mode 3: WTG Power Smoothing

**Figure 25:**
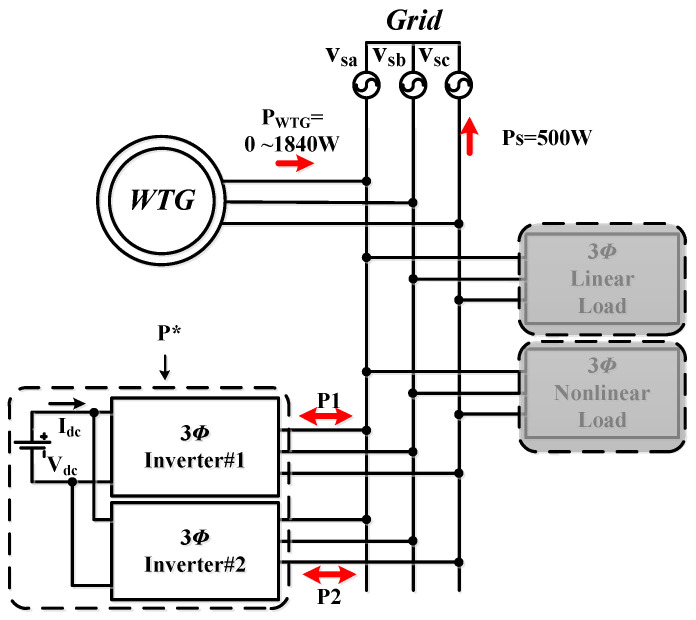
Operating mode 3: operation and power flow.

**Figure 26:**
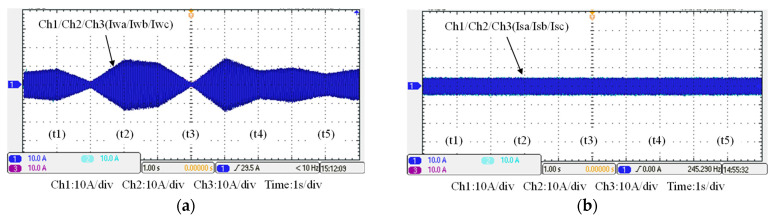

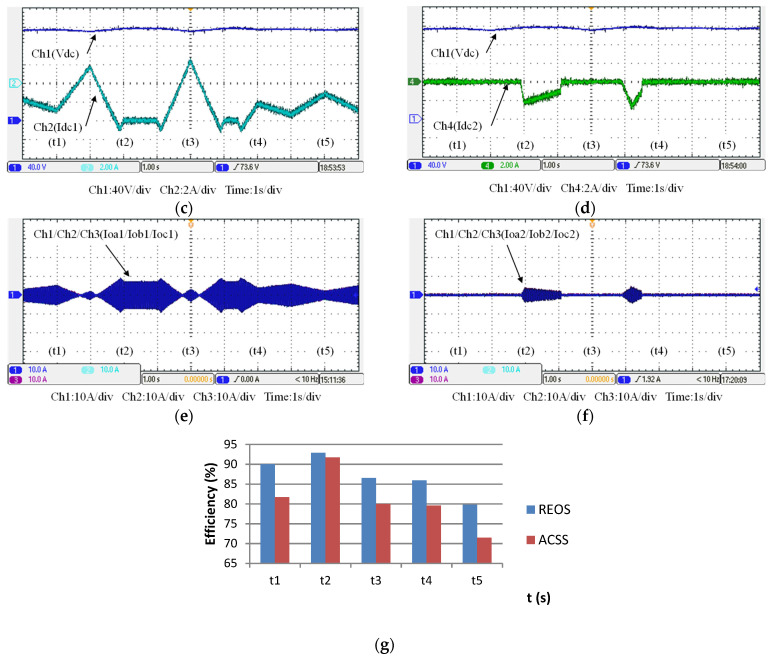
Operating mode 3: (**a**) WTG output currents; (**b**) the grid currents; (**c**) WTGIC’s inverter module #1 DC voltage and current; (**d**) WTGIC’s inverter module #2 DC voltage and current; (**e**) WTGIC’s inverter module #1 AC currents; (**f**) WTGIC’s inverter module #2 AC currents; and (**g**) WTGIC’s efficiency comparison between different control strategies.

This case is implemented to verify the simulation results of WTGIC’s mode 3. In this mode, the WTG power variation range is between 0 W–1840 W, and the target power of the WTG fed into the grid is fixed at 500 W. Figure 25 shows the system operation and power flow of this mode. Typical results are presented in Figure 26.

## 6. Conclusions

In order to buffer the large-scale integration of wind power generation and existing grids in the future, advanced power flow control and compensation for grid-connected WTGs must be developed. This paper has presented a detailed design case of an innovative WTGIC based on parallel-connected three-phase inverters and an ESU and developed an online efficiency improvement algorithm, REOS. The design consideration of the required controllers for the WTGIC based on the dq-axis decoupling method in an SRF has been fully presented. To demonstrate the performance of the proposed REOS and the application potential of the WTGIC, three investigation cases were planned and used for PSIM simulations to validate the proposed algorithm and the WTGIC’s control schemes. Texas Instruments DSP F28335 was used as the control core to carry out the software–hardware integrated implementation of a small-capacity WTGIC experimental system and the complete performance evaluation of the system-wide digital control of all simulated cases. It was found that the simulation and implementation results of the proposed WTGIC working in various application scenarios are in close agreement, and the WTGIC’s efficiency can be improved with the proposed REOS in all cases. The measured average efficiency improvements in the WTGIC in operating modes 1, 2, and 3 were 4.448%, 46.2%, and 6.132%, respectively. These verify the feasibility and effectiveness of the proposed WTGIC and the related control scheme.

## Figures and Tables

**Table 1:** Load specifications in operating mode 2.

Load	Load Value	Active Power Consumption	Reactive Power Consumption
(Case 1) RL_1	12 Ω + 20 mL	723 W	454 Var
(Case 2) RL_2	6 Ω + 10 mL	1445 W	908 Var
(Case 3) Rect_1	36 Ω/7 mH	676 W	48 Var
(Case 4) Rect_2	20 Ω/7 mH	1216 W	423 Var

## Data Availability

No new data were created or analyzed in this study. Data sharing is not applicable to this article.
